# Reproducibility and Relative Validity of a Food Frequency Questionnaire Developed for Female Adolescents in Suihua, North China

**DOI:** 10.1371/journal.pone.0019656

**Published:** 2011-05-11

**Authors:** Wei Xia, Caihong Sun, Li Zhang, Xin Zhang, Jiajia Wang, Hui Wang, Lijie Wu

**Affiliations:** 1 Department of Children Health and Hygiene, School of Public Health, Harbin Medical University, Harbin, China; 2 Department of Nutrition and Food Hygiene, School of Public Health, Harbin Medical University, Harbin, China; Brigham & Women's Hospital, and Harvard Medical School, United States of America

## Abstract

**Background:**

This study aims to evaluate the reproducibility and validity of a food frequency questionnaire (FFQ) developed for female adolescents in the Suihua area of North China. The FFQ was evaluated against the average of 24-hour dietary recalls (24-HRs).

**Methodology/Principal Findings:**

A total of 168 female adolescents aged 12 to 18 completed nine three consecutive 24-HRs (one three consecutive 24 HRs per month) and two FFQs over nine months. The reproducibility of the FFQ was estimated using intraclass correlation coefficients (ICCs), and its relative validity was assessed by comparing it with the 24-HRs. The mean values of the 24-HRs were lower than those of the FFQs, except for protein (in FFQ1) and iron (in FFQ2). The ICCs for all nutrients and food groups in FFQ1 and FFQ2 were moderately correlated (0.4–0.8). However, all the ICCs decreased after adjusting for energy. The weighted κ statistic showed moderate agreement (0.40–0.6) for all nutrients and food groups, except for niacin and calcium, which showed poor agreement (0.35). The relative validity results indicate that the crude Spearman's correlation coefficients of FFQ1 and the 24-HRs ranged from 0.41 (for Vitamin C) to 0.65 (for fruit). The coefficients of each nutrient and food group in FFQ2 and the 24-HRs were higher than those in FFQ1 and the 24-HRs, indicating good correlation. Although all energy-adjusted Spearman's correlation coefficients were lower than the crude coefficients, de-attenuation to correct for intra-individual variability improved the correlation coefficients. The weighted κ coefficients of nutrients and food groups ranged from 0.32 for beans to 0.52 for riboflavin in FFQ1 and the 24-HRs, and 0.32 for Vitamin C to 0.54 for riboflavin in FFQ2 and the 24-HRs.

**Conclusion:**

The FFQ developed for female adolescents in the Suihua area is a reliable and valid instrument for ranking individuals within this study.

## Introduction

Dietary assessment methods should be valid and reliable, and should not impose any burdens on participants and research staff [1]. Among the various instruments designed to assess nutrient and food intake, the food frequency questionnaire (FFQ) is used to estimate individual perception of standard food intake over a defined period (e.g., a year or several months) [2], [3]. Compared with traditional dietary assessment methods, such as the 24-hour dietary recall (24-HR) and dietary records, the FFQ is less expensive and less difficult to accomplish, and easier to administer and evaluate despite the crude information provided [4], [5].

FFQs are widely used throughout the world for epidemiology. Its use for adolescents has increased, but most have been designed for and tested among adolescents in Western countries [6]–[9]. Only a few validation studies on other ethnic groups have been conducted. All FFQs should be tailored to the population of the target market because of the vast difference in food items that contribute to the daily supply of nutrients, as well as the wide range of dietary habits among populations. Food intake also largely varies depending on the ethnic, social, and cultural background of the study population [10].

Suihua is located northeast of China, where the economic condition is not as thriving as that in the southern part of the country. Some chronic nutrition-related diseases, such as iron deficiency or iron deficiency anemia (IDA), are prevalent [11]. Thus, the relationship between diet and anemia was investigated among adolescent girls in Suihua. A new FFQ was designed for adolescent girls, with the specific aim of selecting food groups that provide major contributions to the intake of iron and other nutrients. This study describes the reproducibility of this FFQ and its relative validity against the 24-HRs in female adolescents.

## Methods

### Ethics Statement

This study was approved by the Research Ethics Committee of Harbin Medical University. Written informed consent was obtained from either the participants or their parents (for participants below 18 years old) before they were enrolled in the study.

### Study population

Female adolescents aged 12–18 years were enrolled in the study using a multi-level cluster sampling method. The participants were recruited from three middle schools in the Suihua district of Heilongjiang Province in China. From 200 potential participants, the final sample was narrowed down to 168 subjects. Study participants who did not satisfactorily complete the FFQs (n = 18) or had more than three missing out of the three consecutive 24-HRs (n = 14) were excluded.

### Study design

**Figure 1:**
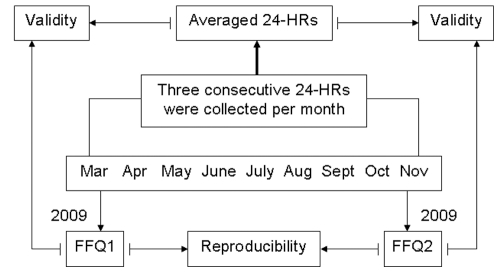
Study design used to test the reliability and relative validity of the food frequency questionnaire.

The study began in March 2009 and continued for the next nine months. Three consecutive 24-HRs were collected from each participant every month. The first FFQ (FFQ1) was administered during the first three consecutive 24-HR, while the second FFQ (FFQ2) was administered in November 2009 during the last three consecutive 24-HR. The study design is shown in Figure 1.

### FFQ and 24-HR

The FFQ was developed in accordance with the methodology proposed by Willett [1]. Revisions ensured that the list of foods reflected the Chinese diet indicated in the 2002 National Health and Dietary Survey [12]. Additional revisions improved estimates of the intake of iron-rich food. Animal food was classified as red meat (livestock meat, animal liver, and blood), white meat (poultry and seafood), eggs, and milk. Food from plants was classified as cereal, vegetable, fruit, and soybean (Table 1). Consumption information across 8 food groups and 86 food items was collected. The respondents were requested to recall the average consumption of a given type of food item during the previous period through a graded scale, with seven levels ranging from never to ≥3 times a day. For seasonal fruits and vegetables, participants were asked to indicate how often these foods were eaten during the season. Portion size was also considered in the survey. Each subject was asked to choose from a set of colored photographs showing different-sized portions of 23 specific food items. Photographs of dishes, bowls, and cups were used to represent the average portion size for the other 63 food items. All FFQ data were double entered, and discrepancies were resolved by referring to the original forms. A nutrient calculator software (Fei Hua V2.3, Institute for Nutrition and Food Security, Chinese Center for Disease Control and Prevention), based on China Food Composition Tables [13], was used by a registered dietician in calculating the daily intakes of calories and nutrients.

**Table 1:** Food grouping used in the study.

Food groups	Food items (n = 86)	Portion size a
Cereal	Steamed bun, bread, corn steamed bum, baked cakeCake, biscuitWheat noodle, fastfood noodle, long rice noodle, corn starch noodleRice, glutinous rice, foxtail millet, purple rice, corn	NumberPieceBowlBowl
Vegetables	Carrot, white radish, soybean sprouts, eggplant, cucumber, pumpkin, hot pepper, Chinese cabbage, spinach, cabbage, celery stem, squash, tomato, potato, onion, chives, parsley, spring onion, cauliflower, garlic flowering stalk, garden pea	Plate
Fruit	Apple, orange, pear, banana, grape, watermelon, peach, casaba, persimmonChinese hawthorn, Chinese pearleaf crab apple, apricotDate, strawberry, cherry	NumberNumberBowl
Red meat	Pork, beef, mutton, pork liver, pork blood, sheep liver, chicken liver, duck blood	Plate
White meat	Chicken, duck, goose meat, cyprinoid fish, morrhua, shrimp, crab, shellfish	Plate
Eggs	Chicken egg, duck egg, pidan, salty egg, goose egg, quail egg	Number
Milk	Milk, yogurt	Cup
Beans	Soybean, small red bean, green gram, black beanSoybean curd, dried bean curd, soybean curd sheet bean productsBean paste, Chinese cheeseSoybean milk	BowlPlateTablespoonCup

a Assigned portion sizes for all 86 food items used in the food frequency questionnaire.

Two dieticians visited each subject nine times to obtain a complete set of data for the three consecutive 24-HR. The interviews were meal sequence-based and covered a detailed assessment and description of the food consumed. The subjects were required to qualitatively and quantitatively describe all food consumed during the previous day by choosing the correct pictures to enable the dietician to estimate food intake. The 24-HR interviews were conducted face-to-face in a classroom on the evening of each visit. The dieticians were instructed to ask the participants key questions for a better qualitative description of food items (e.g., green leafy vegetables, livestock meat, animal liver, blood, etc.). We calculated daily mean intakes of energy, 13 nutrients, and 8 food groups, estimated by nine three consecutive 24-HRs. The mean 24-HR data were used as the standard to measure the relative validity of the FFQ.

### Statistical Analysis

Means and standard deviations (SDs) for energy, nutrients, and food intake were determined for the FFQs and nine three consecutive 24-HRs. The residual method was employed to exclude the possibility of variation due to energy intake ^(1)^. The reproducibility between the first and second FFQ administrations was estimated using the Wilcoxon signed rank test, intraclass correlation coefficient (ICC), weighted kappa (κ) statistic, and misclassification (quartiles method) analyses [14]. The validity of FFQ relative to the 24-HR was assessed by Wilcoxon signed rank test, Spearman's correlation, weighted kappa (κ) statistic, and misclassification (quartiles method) analyses. To take into account within-person variations caused by day-to-day fluctuations and seasonal variations, we “de-attenuated” the Spearman's correlation coefficients using the within- and between-person components of variation from the 24-HRs [15]. The Kruskal-Wallis rank sum test was chosen for multiple comparisons of heme iron/all iron intake ratios on different measured methods.

All statistical analyses were processed using SPSS (Version 13.0) and Excel 2003. Unless otherwise stated, a *P* value <0.05 was considered significant.

## Results

The mean age (SD) and body mass index of the participants were 16.1 (1.3) years and 25.9 (4.5) kg/m^2^, respectively.

The mean intakes of energy, 13 nutrients, and 8 food groups were calculated using the questionnaires as bases and the nutrient calculator software. The comparison of data from the participants is shown in Table 2.

**Table 2:** Daily mean intakes of energy, 13 nutrients, and 8 food groups, estimated by two FFQs and the average of 24-HRs.

Nutrients andfood groups	Mean (SD)		
	24-HRs	FFQ1	FFQ2
Energy (kcal)	1908.71 (321.01)	2166.8 (468.10)	2235.23 (445.21)
Protein (g)	71.54 (25.21)	71.22 (25.67)	82.35 (22.47)
Fat (g)	45.91(23.11)	65.32 (25.33)	59.43 (16.53)
Carbohydrates (g)	302.34 (110.23)	323.51 (121.23)	342.74(131.26)
Fiber (g)	11.87 (11.34)	19.23 (5.41) a	17.85 (7.61) a
Retinol (µg)	535.01 (332.22)	675.34 (323.78)	659.21 (398.23)
Thiamine (mg)	0.86 (0.30)	1.12 (0.34)	1.27(0.21)
Riboflavin (mg)	0.83 (0.21)	1.00 (0.39)	0.91 (0.31)
Niacin (mg)	14.12 (6.18)	21.34 (6.36) a	17.41 (5.31)
Vitamin C (mg)	73.21 (36.33)	143.53 (93.21) a	93.35 (32.57) b
Vitamin E (mg)	17.50 (7.46)	22.57 (9.54)	25.34 (9.83) a
Calcium (mg)	483.64 (231.45)	705.83 (234.05)	605.03 (206.83)
Iron (mg)	24.88 (4.90)	25.42 (6.74)	22.90 (5.93)
Zinc (mg)	11.22 (4.97)	13.74 (2.70)	13.61 (4.62)
Cereal (g)	432.87 (113.57)	461.21 (123.32)	489.35 (158.54)
Vegetables (g)	164.32 (97.68)	197.77 (74.41)	213.63 (103.79)
Fruit (g)	156.35 (87.32)	218.84 (113.23)	195.31 (167.97)
Red meat (g)	37.43 (10.74)	43.25 (27.13)	39.23 (22.05)
White meat (g)	19.15 (5.33)	22.31 (7.02)	19.21 (5.21)
Eggs (g)	27.24 (19.21)	36.24 (21.23)	31.79 (16.95)
Milk (g)	51.23 (17.13)	71.21 (15.33)	59.25 (18.11)
Beans (g)	25.21 (10.46)	28.42 (7.03)	33.32 (17.23) a

a Different from 24-HRs comparing mean values, Wilcoxon signed rank test *P*<0.05.

b Different from FFQ1 comparing mean values, Wilcoxon signed rank test *P*<0.05.

All the mean values of the 24-HRs were lower than those of the FFQs, except for protein (FFQ1) and iron (FFQ2). In FFQ1, the decrements of several nutrients (fiber, niacin, Vitamin C, and calcium) were statistically significant (*P*<0.05). Compared with those of the 24-HRs, the mean values of FFQ2 were significantly higher for fiber, Vitamin E, and soybeans (*P*<0.05). The mean values of FFQ2 were higher than those of FFQ1 for Vitamin C (*P*<0.05).

Table 2 shows that the average daily intake of iron was 24.88 mg as calculated using the 24-HRs, 25.42 mg using FFQ1, and 22.9 mg using FFQ2. However, the nutrition calculation software showed that heme iron was lower at only 17.3%, accounting for all iron intake in the 24-HRs. Heme iron intake was only 21.1% in FFQ1 and 19.9% in FFQ2. The Kruskal-Wallis rank sum test was the method chosen for multiple comparisons between heme iron and all iron intake ratios; this approach is the most basic compared with different measurement methods. The results showed no significant differences (*P*>0.05).

The crude- and energy-adjusted ICCs in FFQ1 and FFQ2 were calculated to assess the reproducibility of the FFQ (Table 3). All nutrients and foods were moderately correlated (0.4–0.8). After adjusting for energy, all the ICCs decreased. The average crude ICC was 0.59 out of a range of 0.43 (fiber) to 0.74 (riboflavin), while the average energy-adjusted ICC was 0.38 out of a range of 0.32 (Vitamin C) to 0.47 (carbohydrates).

**Table 3:** Crude and energy-adjusted ICCs for nutrient and food group intakes in FFQ1 and FFQ2.

Nutrients andfood groups	ICC (FFQ1 vs FFQ2)	
	Crude a	Energy-adjusted b
Energy (kcal)	0.64	-
Protein (g)	0.59	0.42
Fat (g)	0.43	0.38
Carbohydrates (g)	0.59	0.47
Fiber (g)	0.49	0.33
Retinol (µg)	0.62	0.34
Thiamine (mg)	0.52	0.43
Riboflavin (mg)	0.74	0.44
Niacin (mg)	0.65	0.44
Vitamin C (mg)	0.43	0.32
Vitamin E (mg)	0.53	0.33
Calcium (mg)	0.61	0.45
Iron (mg)	0.62	0.43
Zinc (mg)	0.58	0.37
Cereal (g)	0.62	0.43
Vegetables (g)	0.58	0.41
Fruit (g)	0.59	0.35
Red meat (g)	0.73	0.43
White meat (g)	0.65	0.35
Eggs (g)	0.60	0.43
Milk (g)	0.71	0.40
Beans (g)	0.56	0.36
Mean values	0.59	0.38

a All ICC values were significant (*P*<0.05).

b All ICC values were significant (*P*<0.05), except for fiber, retinol, Vitamins C and E, and fruit.

The degree of misclassification associated with the categorized intakes that were assessed using the FFQs was examined as the proportion of participants were classified into *same, adjacent,* and *opposite quartile* (Table 4). The proportion of subjects classified within one quartile (in the same and adjacent categories) by both FFQs ranged from 70.8% (for retinol) to 92.9% (for eggs). Extreme misclassification into opposite quartiles was observed for all nutrients and food groups less than 8%. Higher values were observed for retinol and niacin at 7.8 and 6.0%, respectively. The weighted κ statistic showed moderate conformity, ranging from 0.40 to 0.6 for all nutrients and food groups, except niacin and calcium, which fared poorly at 0.35.

**Table 4:** Comparison and weighted κ of adjusted daily mean intakes of food groups and nutrients based on FFQs.

Nutrients andfood groups	FFQ1 vs FFQ2			
	Same category (%)	Adjacent category (%)	Extreme category (%)	Weightedκ^a^
Energy (kcal)	78.6	6.0	0.6	0.53
Protein (g)	61.3	19.0	4.2	0.47
Fat (g)	58.3	20.8	5.4	0.45
Carbohydrates (g)	74.4	9.5	3.0	0.41
Fiber (g)	55.4	26.8	4.2	0.50
Retinol (µg)	64.3	6.5	7.8	0.45
Thiamine (mg)	73.8	8.9	4.8	0.49
Riboflavin (mg)	72.0	7.7	3.0	0.43
Niacin (mg)	69.6	7.7	6.0	0.35
Vitamin C (mg)	64.9	12.5	1.8	0.41
Vitamin E (mg)	70.2	8.9	3.0	0.45
Calcium (mg)	78.0	11.3	4.8	0.35
Iron (mg)	73.3	11.3	3.6	0.40
Zinc (mg)	83.9	6.5	3.0	0.41
Cereal (g)	78.0	3.0	1.8	0.43
Vegetables (g)	75.6	5.4	1.2	0.42
Fruit (g)	70.2	8.9	4.2	0.51
Red meat (g)	72.0	7.7	3.0	0.47
White meat (g)	64.9	10.1	5.4	0.41
Eggs (g)	83.9	9.0	1.8	0.41
Milk (g)	78.6	7.7	1.8	0.45
Beans (g)	72.0	8.3	4.8	0.45
Mean values	71.5	10.2	3.6	0.42

a All weighted κ coefficients were significant (*P*<0.05), except for niacin and calcium.

The crude, energy-adjusted, and de-attenuated Spearman's correlation coefficients of the FFQs (FFQ1, FFQ2, and averaged FFQ) and the averages resulting from the nine three consecutive 24-HRs are presented in Table 5. These values enable the assessment of the relative validity of the FFQ. The crude Spearman's correlation coefficients of FFQ1 and the 24-HRs ranged from 0.41 (for Vitamin C) to 0.65 (for fruit) with a mean value of 0.54. The energy-adjusted coefficients ranged from 0.32 (for Vitamin E) to 0.46 (for riboflavin) with a mean of 0.39, while the de-attenuated coefficients ranged from 0.44 (for fat and Vitamin C) to 0.66 (for milk) with a mean of 0.51. The coefficients for each nutrient and food group in FFQ2 and the 24-HRs were higher than those in FFQ1 and the 24-HRs. All crude coefficients, whether those of FFQ1 and the 24-HRs or FFQ2 and the 24-HRs, were greater than 0.4, indicating good correlation. All energy-adjusted Spearman's correlation coefficients were lower than crude coefficients in terms of ICCs in FFQ1 and FFQ2. However, de-attenuation to correct for intra-individual variability improved the Spearman's correlation coefficients and led to changes in mean values in FFQ1 and the 24-HRs (0.52 to 0.56) and in FFQ2 and the 24-HRs (0.58 to 0.60).

**Table 5:** Spearman's correlation coefficient of daily mean nutrient and food group intakes obtained from FFQs and the average of 24-HRs.

Nutrients andfood groups	FFQ1 vs 24-HRs			FFQ2 vs 24-HRs			Mean (FFQ1,FFQ2) vs 24-HRs		
	Crude a	Energy-Adjusted b	De-attenuated	Crude a	Energy-Adjusted c	De-attenuated	Crude a	Energy-Adjusted a	De-attenuated
Energy (kcal)	0.64	--	0.63	0.65	--	0.63	0.65	--	0.61
Protein (g)	0.58	0.42	0.59	0.59	0.39	0.60	0.58	0.49	0.61
Fat (g)	0.42	0.39	0.44	0.62	0.43	0.62	0.59	0.53	0.65
Carbohydrates (g)	0.56	0.44	0.59	0.63	0.42	0.65	0.63	0.48	0.63
Fiber (g)	0.49	0.33	0.49	0.52	0.35	0.56	0.53	0.38	0.55
Retinol (µg)	0.44	0.36	0.47	0.62	0.43	0.60	0.60	0.51	0.53
Thiamine (mg)	0.51	0.46	0.53	0.56	0.43	0.58	0.56	0.46	0.57
Riboflavin (mg)	0.48	0.46	0.49	0.73	0.46	0.70	0.63	0.51	0.60
Niacin (mg)	0.63	0.44	0.65	0.66	0.37	0.65	0.67	0.43	0.64
Vitamin C (mg)	0.41	0.33	0.44	0.43	0.44	0.46	0.43	0.40	0.49
Vitamin E (mg)	0.50	0.32	0.54	0.53	0.35	0.55	0.51	0.41	0.55
Calcium (mg)	0.58	0.42	0.60	0.64	0.42	0.65	0.63	0.41	0.58
Iron (mg)	0.53	0.42	0.55	0.62	0.40	0.58	0.59	0.43	0.63
Zinc (mg)	0.54	0.37	0.51	0.62	0.37	0.59	0.59	0.40	0.64
Cereal (g)	0.64	0.41	0.65	0.65	0.46	0.61	0.65	0.43	0.61
Vegetables (g)	0.62	0.44	0.63	0.57	0.44	0.59	0.61	0.51	0.55
Fruit (g)	0.65	0.34	0.62	0.71	0.38	0.70	0.70	0.53	0.63
Red meat (g)	0.55	0.46	0.53	0.62	0.40	0.65	0.59	0.45	0.63
White meat (g)	0.52	0.45	0.53	0.58	0.40	0.41	0.57	0.41	0.61
Eggs (g)	0.54	0.34	0.56	0.61	0.35	0.63	0.59	0.39	0.63
Milk (g)	0.64	0.43	0.66	0.55	0.38	0.57	0.56	0.45	0.53
Beans (g)	0.54	0.36	0.51	0.59	0.40	0.63	0.57	0.40	0.60
Mean values	0.52	0.38	0.56	0.58	0.39	0.60	0.57	0.43	0.59

a All Spearman's correlation coefficients were significant (*P*<0.05).

b All Spearman's correlation coefficients were significant (*P*<0.05), except for fiber, Vitamins C and E, fruit, and eggs.

c All Spearman's correlation coefficients were significant (*P*<0.05), except for fiber, Vitamin E, and eggs.

The classification in quartiles (Table 6) yielded similar results for both FFQs with an average of more than 80% of the subjects classified in the same or adjacent quintiles. The weighted κ coefficients for nutrients and food groups of the FFQs and the 24-HRs are also shown in this table. FFQ1 values ranged from 0.32 (for beans) to 0.52 (for riboflavin), with an average of 0.42. The weighted κ value for FFQ2 also ranged from 0.32 (for Vitamin C) to 0.54 (for riboflavin), with an average value of 0.43. The lowest value for the averaged FFQ was 0.32 (for Vitamin C) and the highest was 0.54 (for both riboflavin and calcium), with an average value of 0.43.

**Table 6:** Comparison and weighted κ of adjusted daily mean intakes of food groups and nutrients based on the average of 24-HRs.

Nutrients andfood groups	FFQ1 vs 24-HRs^a^				FFQ2 vs 24-HRs^b^				Mean FFQ vs 24-HRs c
	Samecategory (%)	Adjacentcategory (%)	Extremecategory (%)	Weightedκ^a^	Samecategory (%)	Adjacentcategory (%)	Extremecategory (%)	Weightedκ^a^	Weightedκ^a^
Energy (kcal)	85.7	4.8	1.2	0.42	73.2	7.7	8.9	0.39	0.41
Protein (g)	88.1	11.3	2.9	0.42	87.5	2.9	5.9	0.48	0.45
Fat (g)	89.9	1.8	1.8	0.41	90.5	3.0	1.8	0.52	0.49
Carbohydrates (g)	86.3	11.9	0	0.41	88.7	10.7	5.9	0.43	0.43
Fiber (g)	85.1	8.9	1.8	0.39	88.7	5.9	1.8	0.41	0.40
Retinol (µg)	88.1	4.1	0	0.39	84.5	4.7	5.9	0.45	0.43
Thiamine (mg)	87.9	7.7	3.0	0.41	86.2	10.1	2.4	0.42	0.42
Riboflavin (mg)	79.1	11.3	0	0.52	84.5	6.5	3.0	0.54	0.54
Niacin (mg)	72.0	22.6	0.6	0.41	83.9	2.9	7.8	0.38	0.40
Vitamin C (mg)	80.3	14.3	402	0.37	69.6	13.7	5.9	0.32	0.32
Vitamin E (mg)	76.2	14.8	6.5	0.37	81.5	8.9	1.2	0.40	0.39
Calcium (mg)	83.9	10.1	2.3	0.46	83.3	7.7	1.2	0.42	0.54
Iron (mg)	81.5	10.7	3.0	0.47	88.7	5.9	2.3	0.48	0.48
Zinc (mg)	84.5	10.1	0.6	0.48	83.3	8.9	1.8	0.45	0.46
Cereal (g)	84.5	2.4	0	0.47	86.3	5.4	0.6	0.51	0.48
Vegetables (g)	76.2	18.5	1.2	0.43	83.9	1.8	5.4	0.41	0.43
Fruit (g)	70.1	19.0	3.0	0.35	83.9	2.9	1.2	0.42	0.40
Red meat (g)	77.9	8.9	4.2	0.39	86.3	4.2	2.9	0.41	0.40
White meat (g)	82.7	11.9	1.2	0.42	81.5	10.7	3.6	0.39	0.40
Eggs (g)	89.9	4.8	0.6	0.45	82.5	5.9	5.9	0.46	0.45
Milk (g)	77.9	7.7	3.0	0.38	85.1	2.9	1.8	0.43	0.39
Beans (g)	68.5	13.7	6.5	0.32	77.7	8.9	6.5	0.37	0.36
Mean values	81.7	10.5	2.2	0.42	83.7	6.5	3.8	0.43	0.43

a All weighted κ values were significant (*P*<0.05), except for fruit and beans.

b All weighted κ values were significant (*P*<0.05), except for Vitamin C.

c All weighted κ values were significant (*P*<0.05), except for Vitamin C and beans.

## Discussion

The number of food items listed in FFQs tends to vary in terms of importance. A review suggested that the number of items listed ranges from 5 to 350 [16]. The FFQ used in this study, which is composed of 86 food items, is considered to have an optimal number of items. With regard to time frame, varying time intervals between FFQ1 and FFQ2, from 15 days [17] to several years [18], have been reported in previous studies. Reproducibility tests are based on the assumption that diet does not change between two questionnaires; thus, reproducibility may ideally be obtained by two closely administered questionnaires [19]–[22]. In this case, however, subjects would likely remember and repeat their responses. In the present study, FFQ administration was repeated after a nine-month interval because the diet of individuals in Suihua is almost unchanged from March to November because of factors related to the local climate. The repeat administration can reduce the daily and seasonal variations in the study population. However, the time reference can reflect changes in intake caused by seasonality, which may have occurred in this study, possibly lowering true correlations especially for nutrients in which fruits and vegetables are the main source (e.g., Vitamin C and fiber).

In previous studies, the ICCs for nutrient intake generally ranged from 0.4 to 0.8 and 0.3 to 0.8 per food group [23]–[26]. The reproducibility of our instrument is similar because moderate correlations for all nutrients and food groups (0.43 to 0.74 for nutrients and 0.56 to 0.67 for food groups) were obtained. The ICCs ranged from 0.32 to 0.54 for nutrients and 0.37 to 0.51 for food groups after adjustments for energy. In our study, energy adjustment did not improve the correlations for nutrients and food items. According to Willett [1], energy adjustment increases correlation coefficients when the variability of nutrient consumption is related to energy intake. However, correlation coefficients decrease when the variability of nutrient consumption depends on systematic errors of overestimation and underestimation. In the present study, the lower correlations may be explained by an increase in correlated measurement error as a consequence of controlling for total energy intake. In addition, the comparison of average daily food and nutrient intakes derived from FFQ1 and FFQ2, based on joint classification by quartiles and the findings from the weighted κ statistic, showed poor agreement for niacin and calcium (0.35). This result can be partially attributed to the indeterminate description of food intake; unclear descriptions may influence nutrient contents. The nine-month time interval is another possible factor because observed reproducibility may be lower than the true value as differences in responses may reflect true changes in dietary habits as well as variations in responses [27].

A major component of the validation process is selecting the appropriate reference method by which to assess the test measurement. No gold standard exists for dietary intake measurement, but it is crucial for the errors of both the methods used in the current study to be as independent of each other as possible. In a review on the validation of FFQs, the authors showed that 75% of the studies validated the FFQs against repeated 24-HRs [16]. Correlations ranging from 0.45 to 0.70 were evident among validation studies on dietary questionnaires [28], [29]. The results of an evaluation of relative validity depend on several factors, including choice of reference method, degree of homogeneity of the intake values within the population, recall period, and the number of days of record collection [30]. In our study, relative validity was tested by comparing FFQ1 and FFQ2 with the average of the nine three consecutive 24-HRs. Good correlations were obtained, especially for the second questionnaire, in which the recalls were generally better. This result may have been caused by a learning effect in the second questionnaire [31]. The reference method used in our study was the average of the nine three consecutive 24-HRs over nine months. Our study population was a group of female adolescents with similar lifestyles (students in three middle schools who often dine in the school cafeteria), which may have contributed to the moderate correlations. The ability to understand abstract concepts and form mental images of one's diet that are as close as possible to actual situations [32] is another crucial factor in producing reliable estimates of habitual intake using the FFQ. Colored photographs of food taken in classrooms were used as a picture-sorting technique designed to make the task less tedious and encourage the interviewees to provide more accurate dietary information [33].

In this study, the average nutrient intake values were lower for the 24-HR data compared with the average values of the FFQs. Most daily intake values of nutrients from different measures are similar to the standards indicated in the Dietary Guidelines for the Chinese population [34], except for calcium, which was lower than the recommended criterion (1,000 mg per day for female adolescents). Although the daily average intake of iron from the FFQs and the 24-HRs satisfied the Dietary Guidelines, the nutrition calculation software showed that the intakes of heme iron were still lower in Suihua female adolescents. Thus, this result can also explain the high prevalence of IDA in the Chinese population [12]. The results also showed an excessive intake of fat and Vitamin E, and an insufficient intake of vegetables, meat (red meat, poultry, fish, and shrimp), and milk. Although this result may be inaccurate, it may serve as reference for the evaluation of the habitual food intake of female adolescents.

The first limitation of this study is that the food groups designed did not include beverages, which may influence energy intake. Typically, the beverage consumption of Chinese female adolescents, especially girls living in a poverty-stricken, cold area like Suihua, is much lower than that of Western females. Another limitation is the small sample used. However, every step of the sampling was randomly performed so that the participants were representative of all teenage girls in the district.

In summary, our dietary assessment is both reproducible and valid, with the observed correlations similar to those reported by other cohort studies. This study suggests that the FFQ can measure the standard intake of major nutrients for female adolescents living in Suihua, North China. Whether this instrument can assess relationships between diet and disease in Chinese adolescent girls will be addressed in future work.
